# Supplementary material for: Endocytic vesicles act as vehicles for glucose uptake in response to growth factor stimulation
Source: Nat Commun. 2024 Apr 2;15:2843. doi: 10.1038/s41467-024-46971-9 (PMC10987504; doi:10.1038/s41467-024-46971-9)
Supplement: Supplementary file 1 — Supplementary Information [file 41467_2024_46971_MOESM1_ESM.pdf]

Supplementary Information for;

Endocytic vesicles act as vehicles for glucose uptake in response to growth factor stimulation

Ryouhei Tsutsumi*, Beatrix Ueberheide, Feng-Xia Liang, Benjamin G. Neel, Ryuichi Sakai, Yoshiro Saito

*Corresponding author. Email: tsutsumi.ryohei@kitasato-u.ac.jp

Supplementary Figs 1-11

Supplementary Fig. 1

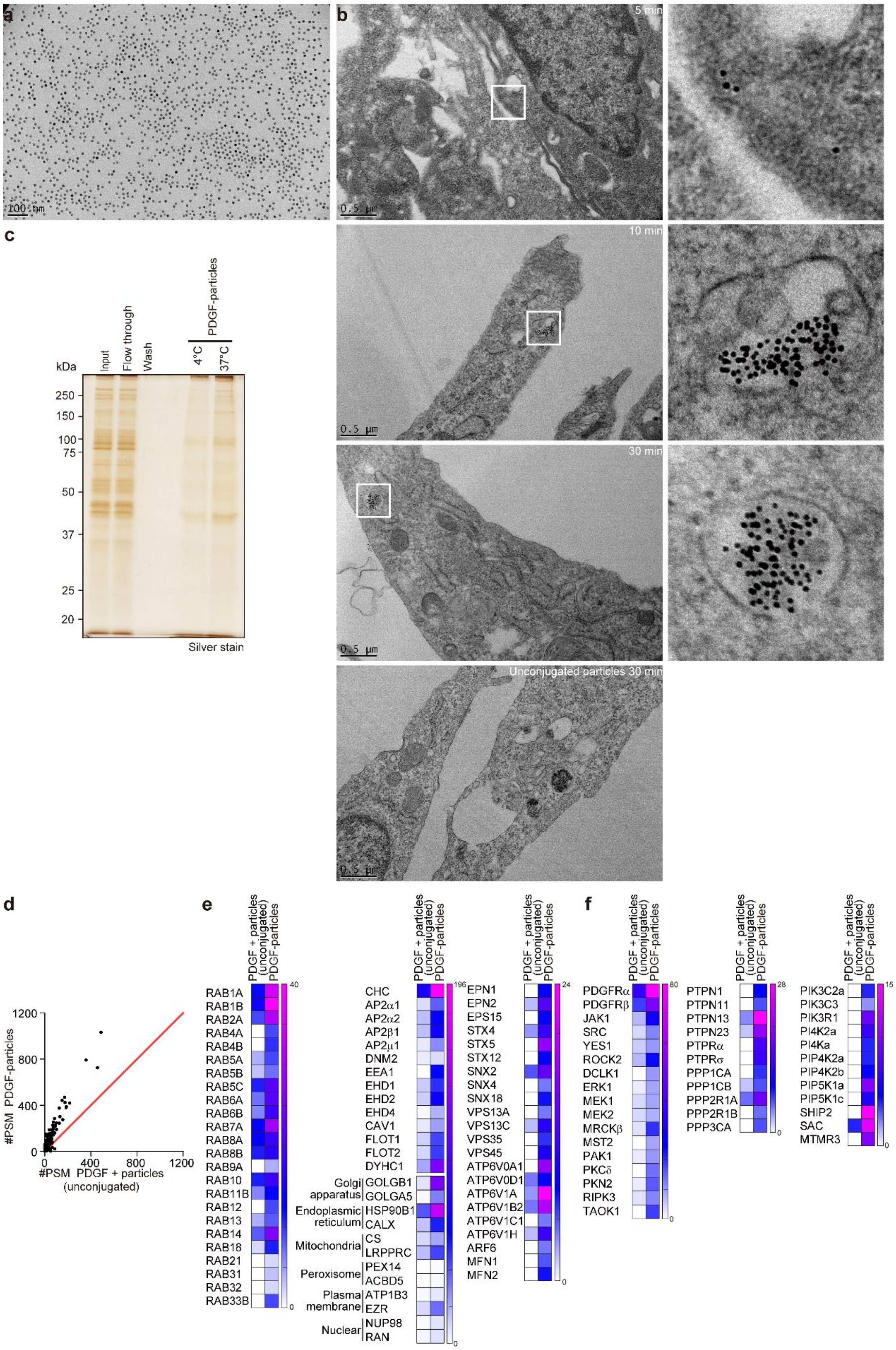

Supplementary Fig. 1. Isolation of PDGFR endocytic vesicles utilizing magnetic nanoparticles.

a. Electron microscopic image of the nanoparticles used in this research. Scale bar: 100 nm. Data were obtained from a single experiment. **b.** Serum-starved Swiss 3T3 fibroblasts were treated with PDGF-BB-conjugated or unconjugated nanoparticles for the indicated times. Cells were fixed and analyzed by transmission electron microscopy. Higher magnification images of the boxed region are shown on the right. Scale bars: 0.5 μm. Data was obtained from a single experiment. **c.** Serum-starved Swiss 3T3 cells were treated with PDGF-BB-conjugated nanoparticles for 5 min at 4°C or 37°C. Post-nuclear supernatants were prepared and subjected to magnetic isolation, SDS-PAGE, and silver staining. Representative data from one of 2 independent experiments are shown. **d-f.** Fractions magnetically isolated from post-nuclear supernatants of PDGF-BB-biotin-conjugated nanoparticle-treated (PDGF-particle) or unconjugated PDGF-BB plus nanoparticle-treated (control) Swiss 3T3 fibroblasts were analyzed by LC-MS/MS. **d.** Proteins identified by LC-MS/MS (see also Table S1) were plotted according to #PSM in the control and the PDGF-particle endocytic vesicle fractions. The red line indicates a 1:1 ratio between the fractions. **e,f.** Heat maps showing #PSMs of the indicated proteins in PDGF plus unconjugated particles (control) and PDGF-particle endocytic vesicle fractions. Data were obtained from a single experiment.

Supplementary Fig. 2

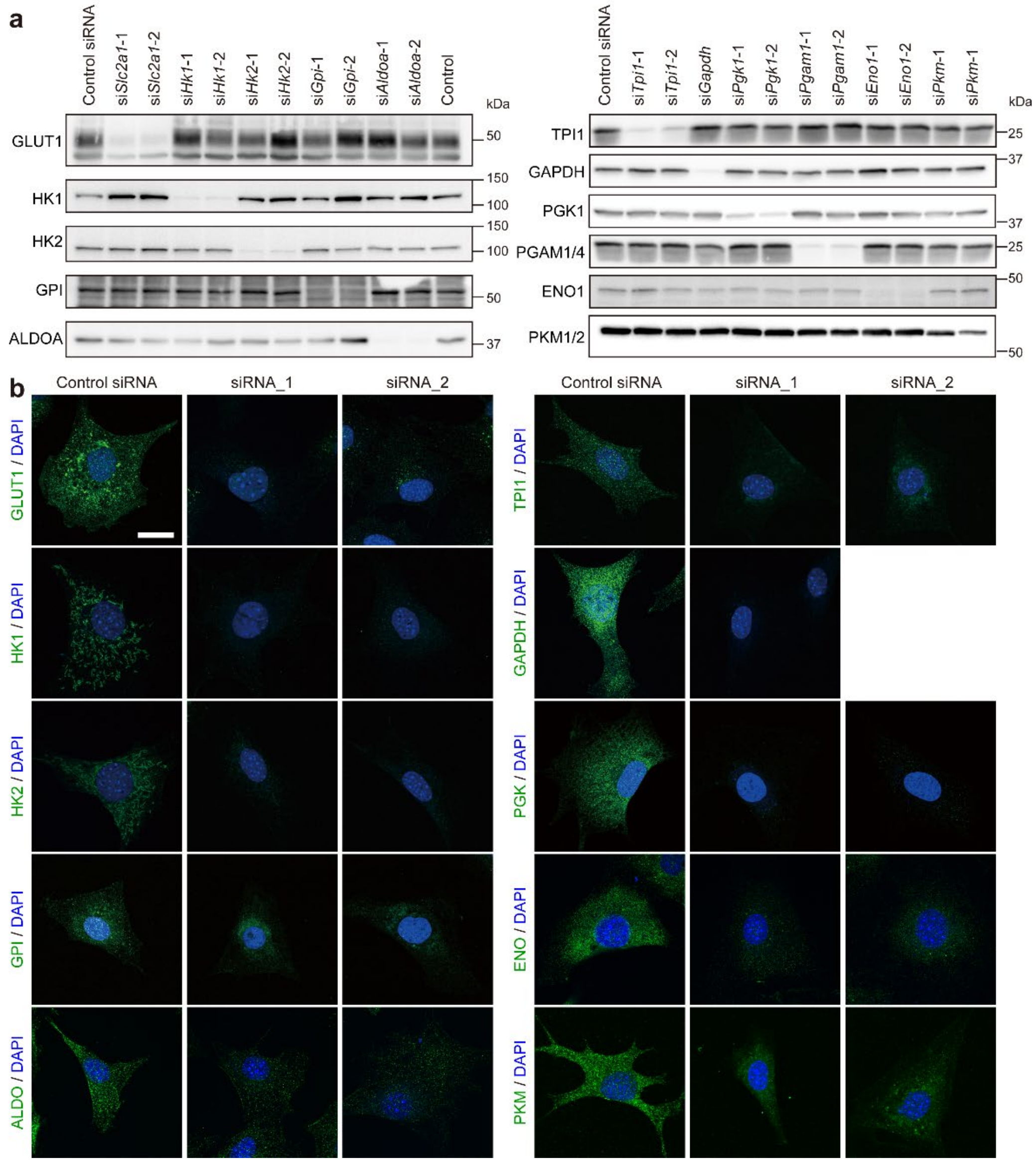

Supplementary Fig. 2. Validation of antibodies against glycolytic enzymes used in this study. **a.** Swiss 3T3 fibroblasts were transfected with the indicated siRNAs and cultured for 72 h. Lysates were subjected to immunoblotting with the indicated antibodies. Representative data are shown from one of 2 independent experiments. **b.** Swiss 3T3 fibroblasts were transfected with the indicated siRNAs and cultured for 72 h. Cells were then immunostained with the indicated antibodies (green). Nuclei were stained with DAPI (blue). Scale bar: 20 µm. Representative data are shown from one of 2 independent experiments. Source data are provided as a Source Data file.

Supplementary Fig. 3

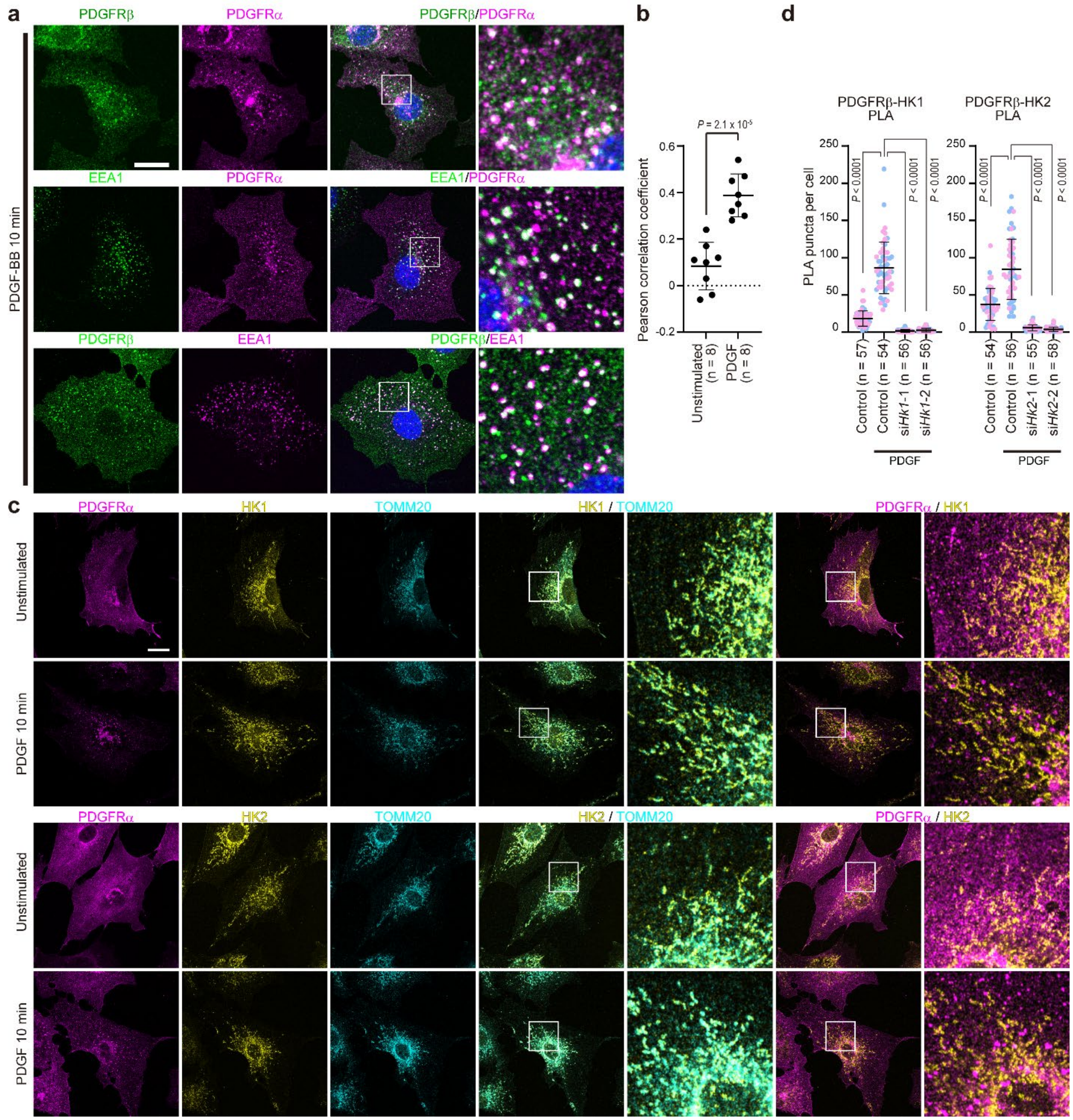

Supplementary Fig. 3. Subcellular localization of GLUT1 and glycolytic enzymes in PDGF-treated cells.

a. Serum-starved Swiss 3T3 fibroblasts were stimulated with 50 ng/ml PDGF-BB for 10 min and subjected to immunofluorescence staining with the indicated antibodies. Nuclei were stained with DAPI (blue). Higher magnification images of the boxed region are shown on the right. Scale bars: 20 μm. Representative data are shown from one of 2 independent experiments. **b.** PDGF-dependent colocalization of PDGFRα and GLUT1 shown in Fig. 2A was quantified. Pearson correlation coefficients were calculated (n = 8 images) and plotted in

the graph. Bars represent mean ± SD. P value was calculated using two-tailed unpaired *t* test. Representative data are shown from one of 2 independent experiments. **c.** Serum-starved Swiss 3T3 fibroblasts were stimulated with 50 ng/ml PDGF-BB for 10 min or left untreated and then subjected to immunofluorescence staining with anti-PDGFRα (magenta), anti-HK1 or anti-HK2 (yellow), and anti-TOMM20 (cyan) antibodies. Higher magnification images of the boxed region are shown as indicated. Scale bars: 20 μm. Representative data are shown from one of 2 independent experiments. **d.** Serum-starved Swiss 3T3 fibroblasts transfected with indicated siRNAs were stimulated with 50 ng/ml PDGF-BB for 10 min and then were subjected to PLA with anti-PDGFRβ and anti-HK1 or anti-HK2 antibodies. PLA signals in the indicated number of cells were counted and plotted in the graphs. Bars and error bars in the graphs represent mean and ± SD of PLA signals per cell. Colors of dots in the graph represent 2 independent biological repeats. P values were calculated using Brown-Forsythe and Welch one-way ANOVA test with post-hoc Games-Howell test. Source data are provided as a Source Data file.

Supplementary Fig. 4

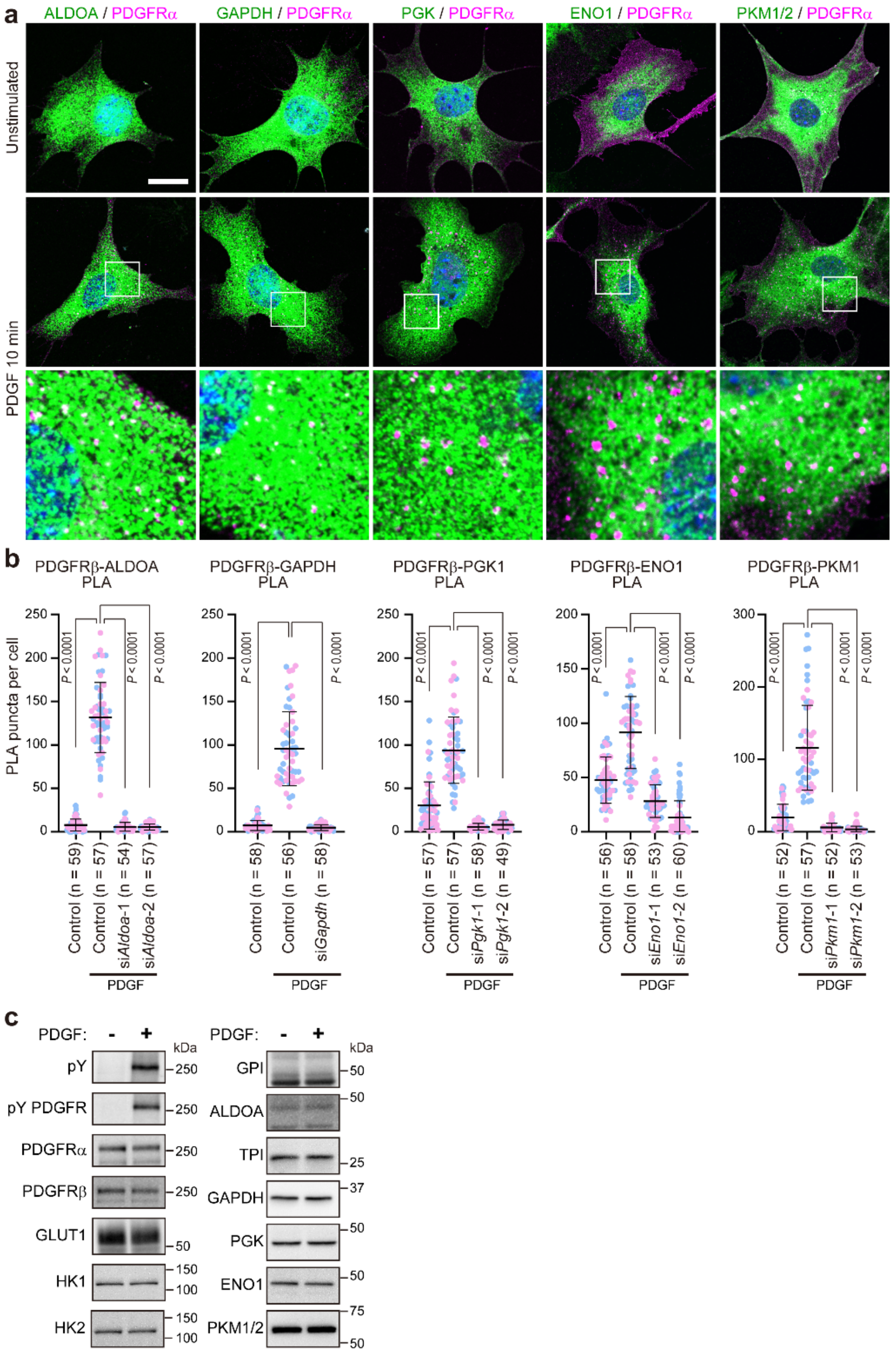

Supplementary Fig. 4. Subcellular localization of glycolytic enzymes in PDGF-treated cells.

a. Serum-starved Swiss 3T3 fibroblasts were stimulated with 50 ng/ml PDGF-BB for 10 min or untreated and subjected to immunofluorescence staining with the indicated antibodies. Nuclei were stained with DAPI (blue). Higher magnification images of the boxed region are shown at the bottom. Scale bars: 20 μm. Representative data are shown from one of 2 independent experiments. **b.** Serum-starved Swiss 3T3 fibroblasts transfected with indicated siRNAs were stimulated with 50 ng/ml PDGF-BB for 10 min and then were subjected to PLA with anti-

PDGFRβ and indicated antibodies. PLA signals in the indicated number of cells were counted and plotted in the graphs. Bars and error bars in the graphs represent mean and ± SD of PLA signals per cell. Colors of dots in the graph represent independent 2 biological repeats. P values were calculated using Brown-Forsythe and Welch one-way ANOVA test with post-hoc Games-Howell test. **c.** Serum-starved Swiss 3T3 fibroblasts were stimulated with or without 50 ng/ml PDGF-BB for 10 min. Lysates were subjected to immunoblotting with the indicated antibodies. Representative data are shown from one of 2 independent experiments. Source data are provided as a Source Data file.

Supplementary Fig. 5

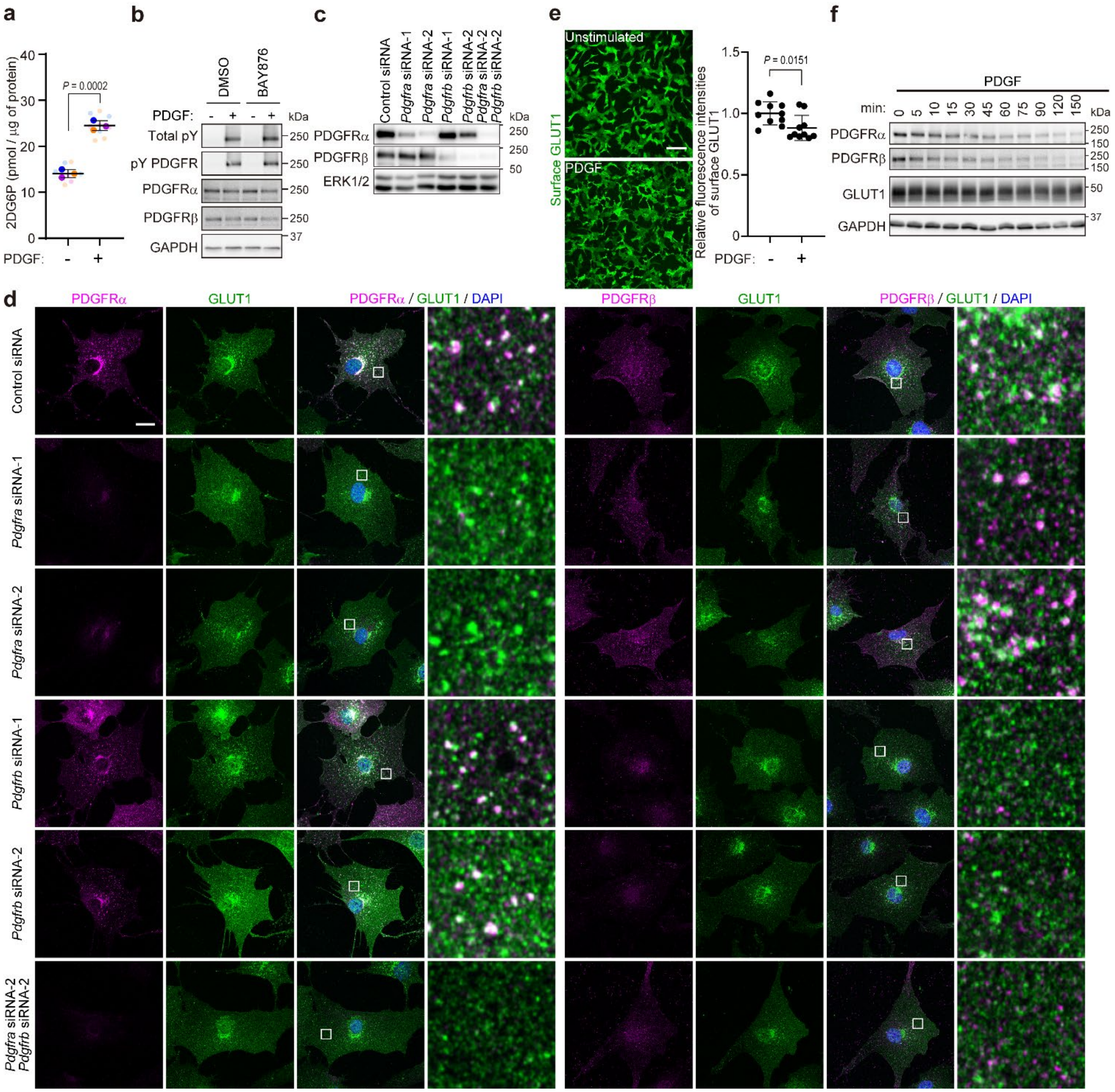

Supplementary Fig. 5. PDGF-evoked cellular glucose uptake and receptor endocytosis.

a. Serum-starved Swiss 3T3 fibroblasts were subjected to glucose uptake assay with or without PDGF-BB (50 ng/ml) in the presence of 2DG for 10 min. Graph shows 2DG6P normalized to total cellular proteins. Bars and error bars in the graph show means of 3 independent biological replicates and ± SD (biological replicates, n = 3). Values of biological replicates (deep-color dots) indicate means of technical replicates (light-color dots). Colors of dots represent each biological replicate. P values were calculated using unpaired two-tailed *t* test. **b.** Serum-starved Swiss 3T3 fibroblasts were pre-treated with BAY876 (50 nM) for 20 min or left untreated, and then were

stimulated with PDGF-BB for 10 min or left untreated, as shown in Fig. 3A. Lysates were subjected to immunoblotting with indicated antibodies. Representative data are shown from one of 2 independent experiments. **c.** Lysates from Swiss 3T3 fibroblasts transfected with indicated siRNAs were subjected to immunoblotting with the indicated antibodies. Representative data are shown from one of 2 independent experiments. **d.** Serum-starved Swiss 3T3 fibroblasts transfected with indicated siRNAs were stimulated with 50 ng/ml PDGF-BB for 10 min and subjected to immunofluorescence staining with anti-PDGFRα or anti-PDGFRβ (magenta) and anti-GLUT1 (green) antibodies. Nuclei were stained with DAPI (blue). Higher magnification images of the boxed region are shown on the right. Scale bars: 20 μm. Representative data are shown from one of 2 independent experiments. **e.** Cell surface GLUT1 was labeled (green) in serum-starved or PDGF-stimulated Swiss 3T3 fibroblasts. Scale bar: 100 μm. Graph shows average fluorescence intensity per cell area in images (10 images each, technical replicates, n = 10), relative to the average in unstimulated cells (normalized to 1). Error bars represent ± SD. P values were calculated by unpaired two-tailed *t* test. Representative data are shown from one of 2 independent experiments. **f.** Serum-starved Swiss 3T3 fibroblasts were stimulated with PDGF-BB (50 ng/ml) for the indicated times. Lysates were subjected to immunoblotting with the indicated antibodies. Representative data are shown from one of 2 independent experiments. Source data are provided as a Source Data file.

Supplementary Fig. 6

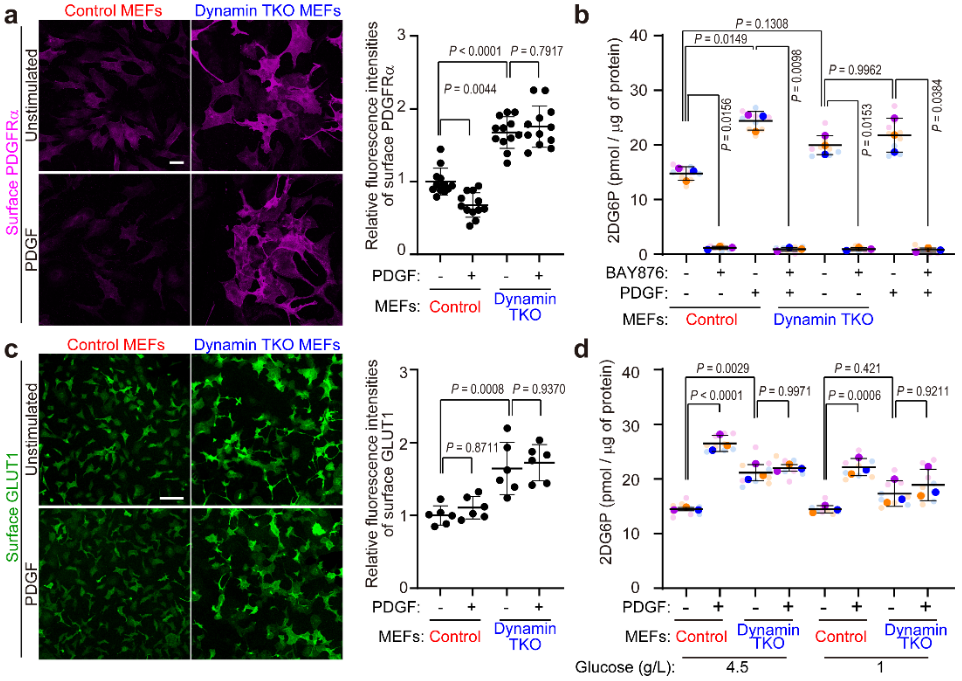

Supplementary Fig. 6. Requirement for receptor endocytosis in growth factor-evoked glycolysis. **a.** Serum-starved control or dynamin TKO MEFs were stimulated with PDGF-BB and subjected to surface PDGFRα staining (magenta). Scale bar: 50 µm. Graph shows fluorescence intensity per cell area (technical replicates, n = 12), relative to unstimulated cells, and ± SD. One-way ANOVA and post-hoc Tukey's test. Representative data are shown from one of 2 independent experiments. **b.** Serum-starved MEFs were treated with BAY876 (50 nM) and subjected to glucose uptake assay. Graph shows 2DG6P normalized to total cellular proteins. Bars and error bars show means of biological replicates (deep-color dots, n = 3) and ± SD. Values of biological replicates are means of technical replicates (light-color dots). One-way ANOVA and post-hoc Tukey's test. **c.** Serum-starved control or dynamin TKO MEFs were stimulated with PDGF-BB for 10 min, followed by labelling of cell surface GLUT1 (green). Scale bar: 100 µm. The graph shows the average fluorescence intensity per cell area (technical replicates, n = 6), relative to the average in unstimulated control cells. Error bars represent ± SD. One-way ANOVA with post-hoc Tukey's test. Representative data are shown from one of 2 independent experiments. **d.** Control or dynamin TKO MEFs were maintained in DMED containing indicated concentrations of glucose for 5 days, serum-starved, and subjected to glucose uptake assay. Graph shows 2DG6P normalized to total cellular proteins. Bars and error bars show means of biological replicates (deep-color dots, n = 3) and ± SD. Values of biological replicates are means of technical replicates (light-color dots). One-way ANOVA and post-hoc Tukey's test. Source data are provided as a Source Data file.

Supplementary Fig. 7

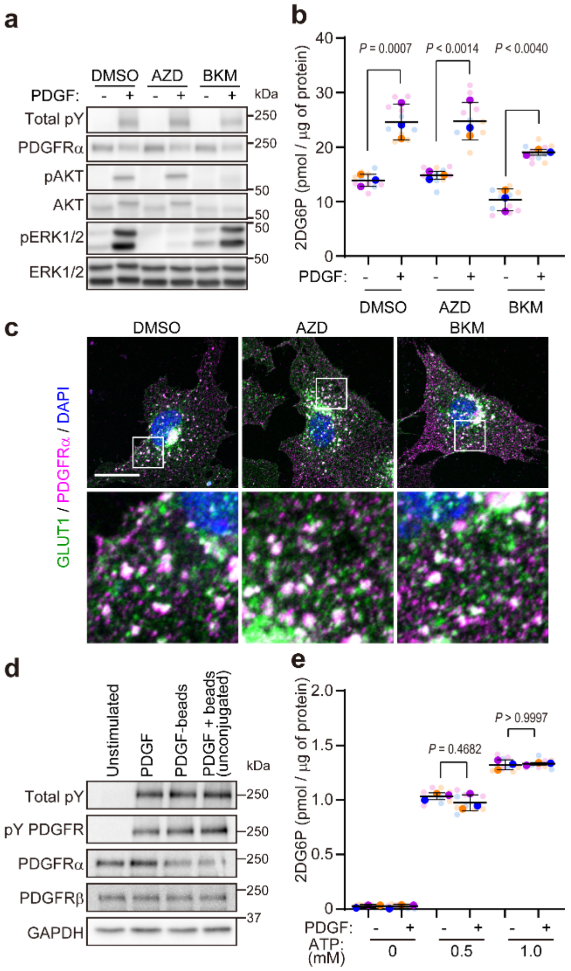

Supplementary Fig. 7. Growth factor-evoked glycolysis depends on receptor endocytosis but not MAPK or PI3K signaling or HK activation.

a-c. Serum-starved Swiss 3T3 fibroblasts were treated with AZD6244 (5 µM) or BKM120 (2.5 µM) and stimulated with PDGF-BB. **a.** Cells were subjected to immunoblotting with indicated antibodies. Representative data are shown from one of 2 independent experiments. **b.** Cells were subjected to glucose uptake assay. Graph shows 2DG6P normalized to total cellular proteins. Bars and error bars show means of biological replicates (deep-color dots, n = 3) and ± SD. Values of biological replicates are means of technical replicates (light-color dots). One-way ANOVA and post-hoc Tukey's test. **c.** Cells were subjected to immunostaining with indicated antibodies. Higher magnification images of the boxed regions are shown. Nuclei were stained with DAPI (blue). Representative data are shown from one of 2 independent experiments each. Scale bar: 20 µm. **d.** Serum-starved Swiss 3T3 fibroblasts were incubated with the indicated stimulation as in Fig. 3F. Lysates were subjected to immunoblotting. Representative data are shown from one of 2 independent experiments. **e.** Serum-starved Swiss 3T3 fibroblasts were stimulated with PDGF-BB and subjected to in vitro hexokinase assay. Graph shows 2DG6P normalized to total cellular proteins. Bars and error bars show means of biological replicates (deep-color dots, n =

3) and ± SD. Values of biological replicates are means of technical replicates (light-color dots). One-way ANOVA and post-hoc Tukey's test. Source data are provided as a Source Data file.

Supplementary Fig. 8

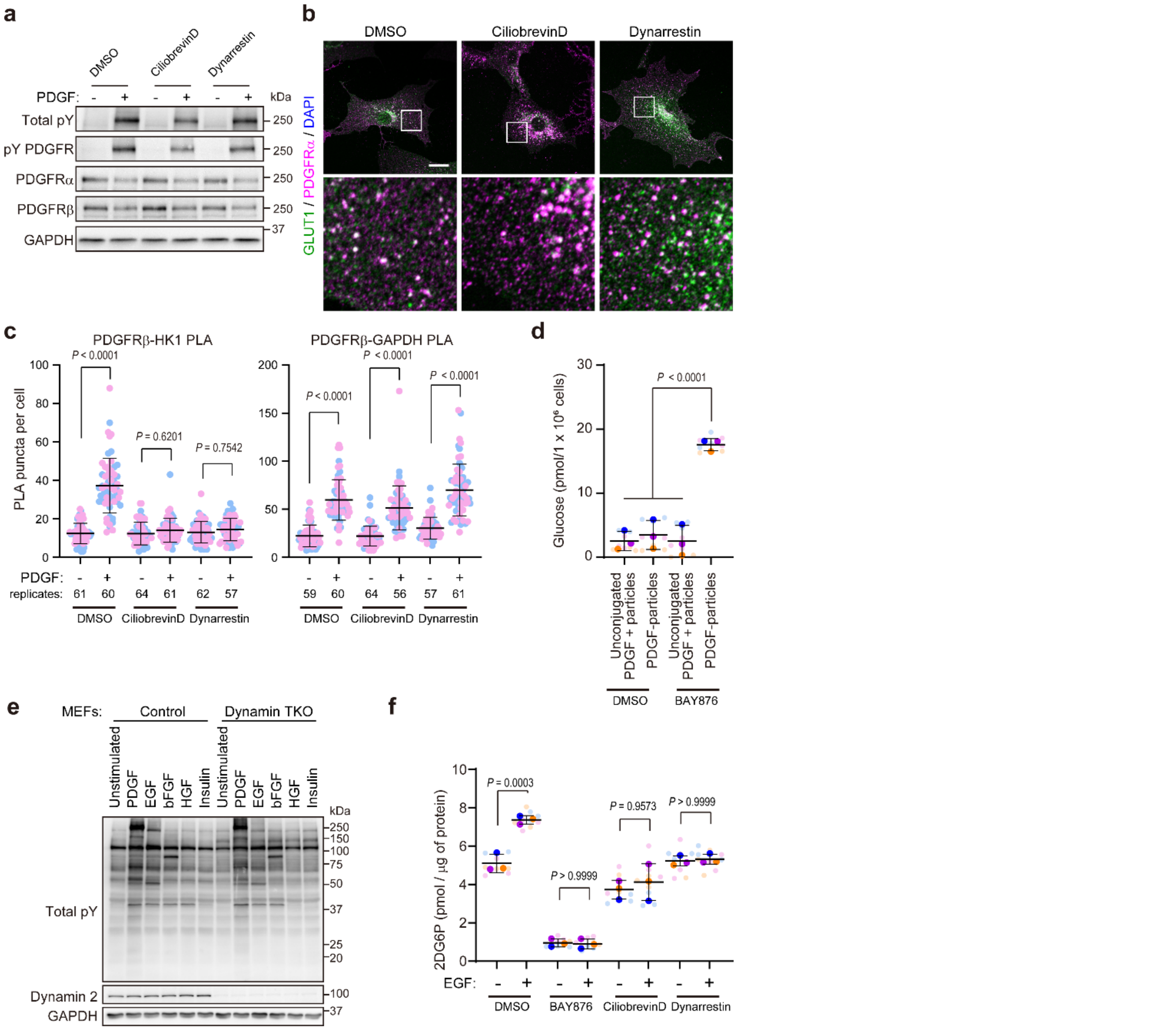

Supplementary Fig. 8. Endocytic vesicles act as vehicles for glucose uptake.

a-c. Serum-starved Swiss 3T3 fibroblasts were treated with Ciliobrevin D (50 µM) or Dynarrestin (50 µM) and stimulated with PDGF-BB as Fig. 3G. **a.** Lysates were subjected to immunoblotting with indicated antibodies. Representative data are shown from one of 2 independent experiments. **b.** Cells were subjected to immunofluorescence staining with indicated antibodies. Higher magnification images of the boxed regions are shown. Representative data are shown from one of 2 independent experiments each. Scale bar: 20 µm. **c.** Cells were subjected to PLA with anti-PDGFRβ and anti-HK1 (right) or anti-GAPDH (left) antibodies. PLA signals in the indicated number of cells were counted and plotted in the graphs. Bars and error bars in the graphs represent mean and ± SD of PLA signals per cell. Colors of dots in the graph represent 2 biological repeats. Brown-Forsythe and Welch one-way ANOVA test with post-hoc Games-Howell test. **d.** Serum-starved Swiss 3T3 cells

were pre-treated with or without BAY876 (50 nM) and treated with PDGF-BB-conjugated nanoparticles or unconjugated PDGF-BB plus nanoparticles for 5 min at 4°C or 37°C. Post-nuclear supernatants were subjected to magnetic isolation in the presence of BAY876 (50 nM) or DMSO. Glucose in the elutes were quantified. Graph shows glucose per 1 x 10^6 cells. Bars and error bars show means of biological replicates (deep-color dots, n = 3) and ± SD. Values of biological replicates are means of technical replicates (light-color dots). One-way ANOVA and post-hoc Tukey's test. **e.** Serum-starved control or dynamin TKO MEFs were stimulated with the indicated growth factors (50 ng/ml) for 10 min. Lysates were subjected to immunoblotting with the indicated antibodies. Representative data are shown from one of 2 independent experiments. **f.** Serum-starved HeLa cells were subjected to glucose uptake assay with or without EGF (50 ng/ml). Graph shows 2DG6P normalized to total cellular proteins. Bars and error bars show means of biological replicates (deep-color dots, n = 3) and ± SD. Values of biological replicates are means of technical replicates (light-color dots). One-way ANOVA and post-hoc Tukey's test. Source data are provided as a Source Data file.

Supplementary Fig. 9

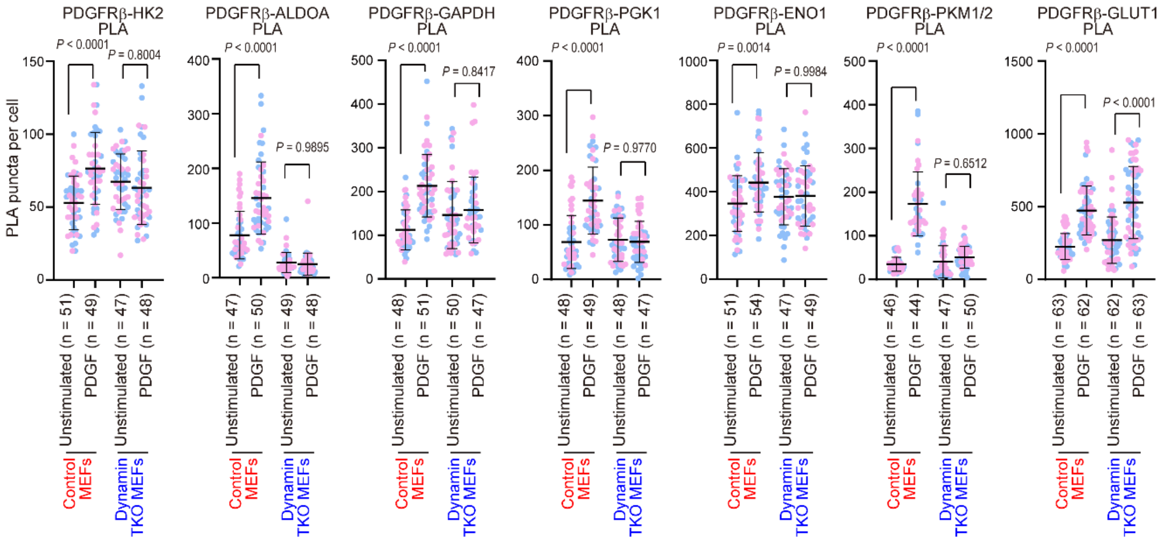

Supplementary Fig. 9. Requirement for dynamin in PDGF-dependent clustering of PDGFRα and glycolytic enzymes.

Serum-starved untreated (control MEFs) or 4-OHT-treated dynamin TKO MEFs were stimulated with 50 ng/ml PDGF-BB for 10 min, and were then subjected to PLA with anti-PDGFRβ and the indicated antibodies. PLA signals in the indicated number of cells were counted and plotted in the graphs. Bars and error bars in the graphs represent mean and ± SD of PLA signals per cell. Colors of dots in the graph represent 2 independent biological repeats. P values were calculated using Brown-Forsythe and Welch one-way ANOVA test with post-hoc Games-Howell tests. Source data are provided as a Source Data file.

Supplementary Fig. 10

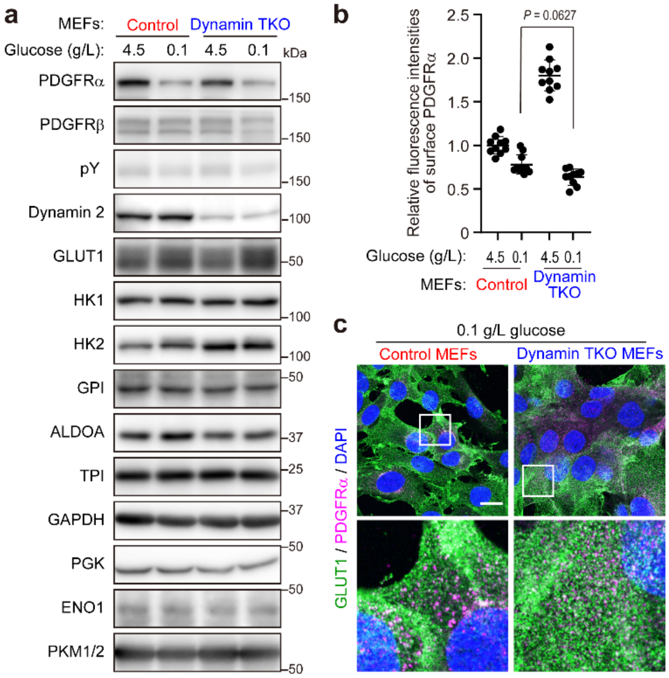

Supplementary Fig. 10. PDGFRs and glycolytic enzymes in glucose limiting condition.

a-c. Conditional Dynamin TKO MEFs treated with 4-OHT (Dynamin TKO) or untreated (control) were cultured in DMEM containing indicated concentrations of glucose without FBS and pyruvate in the presence of PDGF-BB (20 ng/ml) as Fig.4 for 24 h, at the time of metabolomic analysis. 4. **a.** Lysates from the cells were subjected to immunoblotting with indicated antibodies. **b.** Cell were subjected to immunofluorescence staining of surface PDGFRα. Graph shows average fluorescence intensity per cell area in images (10 images each, technical replicates, n = 10), relative to the average in unstimulated cells (normalized to 1). Error bars represent ± SD. P values were calculated by one-way ANOVA and post-hoc Tukey's test. Representative data are shown from one of 2 independent experiments. **c.** Cells were subjected to immunofluorescence staining with anti-GLUT1 (green) and anti-PDGFRα (magenta) antibodies. Nuclei were stained with DAPI (blue). Higher magnification images of the boxed regions are shown at the bottom. Scale bar: 20 μm. Representative data are shown from one of 2 independent experiments. Source data are provided as a Source Data file.

Supplementary Fig. 11

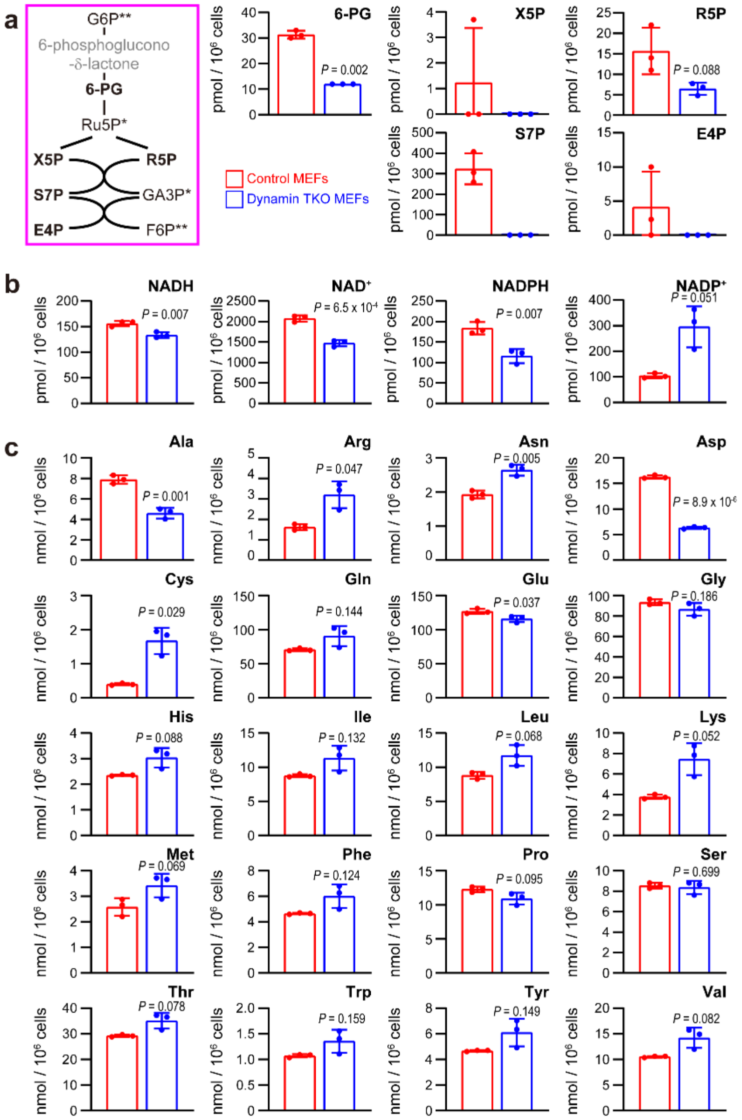

Supplementary Fig. 11. Role of receptor endocytosis in cellular metabolism.

a-c. Graphs show average amounts of each metabolite in 3 samples (technical replicates, n = 3), normalized to cell number. Error bars represent ± SD. P values were calculated using two-tailed Welch's *t* tests. The scheme in **a** shows metabolites in the pentose phosphate pathway. Metabolites not tested are labeled in gray; asterisks indicate metabolites that were not detected in any sample. Data were obtained from a single experiment.
