# Supplementary material for: Endocytic vesicles act as vehicles for glucose uptake in response to growth factor stimulation
Source: Nat Commun. 2024 Apr 2;15:2843. doi: 10.1038/s41467-024-46971-9 (PMC10987504; doi:10.1038/s41467-024-46971-9)
Supplement: Supplementary file 3 — Description of Additional Supplementary Files [file 41467_2024_46971_MOESM3_ESM.pdf]

Description of Additional Supplementary Files

Supplementary Data 1

Proteins in PDGFR-endocytic vesicle fraction detected by MS.

Supplementary Data 2

Metabolomic analysis of fibroblasts in glucose-limiting condition.
