# Supplementary material for: Endocytic vesicles act as vehicles for glucose uptake in response to growth factor stimulation
Source: Nat Commun. 2024 Apr 2;15:2843. doi: 10.1038/s41467-024-46971-9 (PMC10987504; doi:10.1038/s41467-024-46971-9)
Supplement: Supplementary file 6 — Reporting Summary [file 41467_2024_46971_MOESM6_ESM.pdf]

nature portfolio

Corresponding author(s): Ryouhei Tsutsumi

Last updated by author(s): Mar 5, 2024

Reporting Summary

Nature Portfolio wishes to improve the reproducibility of the work that we publish. This form provides structure for consistency and transparency in reporting. For further information on Nature Portfolio policies, see our Editorial Policies and the Editorial Policy Checklist.

Statistics

For all statistical analyses, confirm that the following items are present in the figure legend, table legend, main text, or Methods section.

n/a	Confirmed	
☐	☒	The exact sample size (n) for each experimental group/condition, given as a discrete number and unit of measurement
☐	☒	A statement on whether measurements were taken from distinct samples or whether the same sample was measured repeatedly
☐	☒	The statistical test(s) used AND whether they are one- or two-sided<br>*Only common tests should be described solely by name; describe more complex techniques in the Methods section.*
☒	☐	A description of all covariates tested
☒	☐	A description of any assumptions or corrections, such as tests of normality and adjustment for multiple comparisons
☐	☒	A full description of the statistical parameters including central tendency (e.g. means) or other basic estimates (e.g. regression coefficient) AND variation (e.g. standard deviation) or associated estimates of uncertainty (e.g. confidence intervals)
☐	☒	For null hypothesis testing, the test statistic (e.g. F, t, r) with confidence intervals, effect sizes, degrees of freedom and P value noted<br>*Give P values as exact values whenever suitable.*
☒	☐	For Bayesian analysis, information on the choice of priors and Markov chain Monte Carlo settings
☒	☐	For hierarchical and complex designs, identification of the appropriate level for tests and full reporting of outcomes
☐	☒	Estimates of effect sizes (e.g. Cohen's d, Pearson's r), indicating how they were calculated

Our web collection on statistics for biologists contains articles on many of the points above.

Software and code

Policy information about availability of computer code

Data collection	LC-MS/MS, Proteome Discoverer 1.4 (Thermo)<br>Luminograph I, ImageSaver6 Ver. 2.7.2 (ATTO)<br>Amersham™ ImageQuant™ 800, IQ800 Control Software Ver. 1.2.0 (Amersham)<br>FV1000 confocal microscopy system, FV-10 ASW Ver. 1.6 (Olympus)<br>LSM710 confocal microscopy system, Zen 2.3 Ver. 14.0.23.201 (Zeiss)<br>LSM810 confocal microscopy system, Zen (blue edition) 3.6 (Zeiss)<br>SpectraMaxiD5 and SpectraMaxL, Soft Max Pro 7 Ver. 7.1 (Molecular Devices)<br>CE-TOFMS, Agilent G2201AA ChemStation software Ver. B.03.01 (Agilent) and MasterHands (Keio University)
Data analysis	LC-MS/MS, Proteome Discoverer 1.4 (Thermo)<br>Gene annotation enrichment analysis, DAVID Bioinformatics Resources Ver. 6.8 (LHRI)<br>Image analyses, FIJI/ImageJ Ver. 1.53<br>Statistics, Graphpad Prism 8 Ver. 8.4.3 (GraphPad Software)<br>CE-TOFMS, MassHunter Quantitative Analysis Ver. B.04.00 (Agilent Technologies)

For manuscripts utilizing custom algorithms or software that are central to the research but not yet described in published literature, software must be made available to editors and reviewers. We strongly encourage code deposition in a community repository (e.g. GitHub). See the Nature Portfolio guidelines for submitting code & software for further information.

Data

Policy information about availability of data

All manuscripts must include a data availability statement. This statement should provide the following information, where applicable:

- Accession codes, unique identifiers, or web links for publicly available datasets
- A description of any restrictions on data availability
- For clinical datasets or third party data, please ensure that the statement adheres to our policy

The mass spectrometric raw files for proteomic analysis are accessible at https://massive.ucsd.edu under accession MassIVE MSV000092208 and at www.proteomexchange.org under accession PXD043112. The mass spectrometric raw files for metabolomic analysis are not available due to the inclusion of the service provider's confidential analytical parameters, but the absolute amounts of metabolites calculated are shown in Supplementary Data 2. Other source data are provided with this paper as a Source Data file.

Research involving human participants, their data, or biological material

Policy information about studies with human participants or human data. See also policy information about sex, gender (identity/presentation), and sexual orientation and race, ethnicity and racism.

Reporting on sex and gender	n/a
Reporting on race, ethnicity, or other socially relevant groupings	n/a
Population characteristics	n/a
Recruitment	n/a
Ethics oversight	n/a

Note that full information on the approval of the study protocol must also be provided in the manuscript.

Field-specific reporting

Please select the one below that is the best fit for your research. If you are not sure, read the appropriate sections before making your selection.

☒ Life sciences ☐ Behavioural & social sciences ☐ Ecological, evolutionary & environmental sciences

For a reference copy of the document with all sections, see nature.com/documents/nr-reporting-summary-flat.pdf

Life sciences study design

All studies must disclose on these points even when the disclosure is negative.

Sample size	No statistical method was used to predetermine sample sizes. Sample sizes were determined based on the previous study published by us (Tsutsumi et al, Nat. Commun. 8, 466, 2017) and others using similar biological samples and techniques to detect the significant difference (Avanzato et al, Canceer Res. 78, 3432-3444, 2018; Shimobayashi et al. J. Clin. Invest. 128, 1538-1550, 2018).
Data exclusions	No data was excluded from the analyses.
Replication	Proteomic and metabolomic analyses were conducted as single biological experiments due to limited resources available to the first author. Other data were derived from at least 2 biological repeats, and all repeats were successful. Number of replicates for each experiment is found in the figure legends.
Randomization	Samples were not randomized. In vitro experiments conducted in this study required a priori information of the cells and/or treatment groups, making randomization difficult.
Blinding	The investigators were not blinded to allocation during experiments or outcome assessment. In vitro experiments conducted in this study required a priori information of the cells and/or treatment groups, making blinding difficult.

Reporting for specific materials, systems and methods

We require information from authors about some types of materials, experimental systems and methods used in many studies. Here, indicate whether each material, system or method listed is relevant to your study. If you are not sure if a list item applies to your research, read the appropriate section before selecting a response.

Materials & experimental systems

n/a	Involved in the study	
☐	☒	Antibodies
☐	☒	Eukaryotic cell lines
☒	☐	Palaeontology and archaeology
☒	☐	Animals and other organisms
☒	☐	Clinical data
☒	☐	Dual use research of concern
☒	☐	Plants

Methods

n/a	Involved in the study	
☒	☐	ChIP-seq
☒	☐	Flow cytometry
☒	☐	MRI-based neuroimaging

Antibodies

Antibodies used

Rabbit monoclonal anti-EEA1 (C45B10, #3288, IB 1/1000, IF 1/200), anti-Hexokinase I (C35C4, #2024, IB 1/1000, IF 1/200, PLA 1/200), anti-GAPDH (D16H11, #5174, IB 1/1000, IF 1/200, PLA 1/200), anti-PKM 1/2 (C103A3, #3190, IB 1/1000, IF 1/100, PLA 1/100), anti-PKM2 (D78A4, #4053, IB 1/1000), anti-pY849 PDGFRa/pY857PDGFRb (C43E9, #3170, IB 1/1000), anti-AKT (C67E7, #4691, IB 1/1000), anti-pT308 AKT (D25E6, #13038, IB 1/1000), anti-ERK1/2 (137F5, #4695, IB 1/1000), and anti-phospho ERK1/2 (D13.14.4E, #4370, IB 1/1000) antibodies were purchased from Cell Signaling Technology. Rabbit monoclonal anti-GLUT1 (ab115730, IB 1/1000, IF 1/500, PLA 1/500), anti-Hexokinase II (ab209847, IB 1/1000, IF 1/100, PLA 1/100), anti-PFKL (ab181064, IB 1/1000), anti-PGK1 (ab199438, IB 1/1000, IF 1/250, PLA 1/250), and anti-ENO1/2/3 (ab189891, IB 1/1000, IF 1/500, PLA 1/500) antibodies were from Abcam. Goat polyclonal anti-PDGFRalpha antibodies (AF1062, IB 1/1000, IF 1/200) were purchased from R&D systems. Rabbit polyclonal anti-pS226 GLUT1 antibody (ABN991, IB 1/200) and mouse anti-phosphotyrosine monoclonal antibody cocktail 4G10 Platinum (05-1050, IB 1/1000) were purchased from Millipore. Recombinant polyclonal anti-ALDOA antibody cocktail (711764, IB 1/200, IF 1/100, PLA 1/100) was purchased from Invitrogen. Goat polyclonal anti-PDGFRbeta (sc-1627, IB 1/1000, IF 1/200, PLA 1/200), anti-EEA1 (sc-6414,IF 1/200), anti-clathrin heavy chain (sc-6579, IB 1/1000), anti-dynamin II (sc-6400, IB 1/1000), and rabbit polyclonal anti-PDGFRbeta (sc-432, IF 1/200) antibodies were purchased from Santa Cruz Biotechnology but are currently discontinued. Mouse monoclonal anti-SHP2 (B-1, sc-7384, IB 1/1000), anti-TPI1 (H-11, sc-166785, IB 1/1000, IF 1/200), anti-GPI (H-10, sc-365066, IB 1/1000, IF 1/200), and anti-PGAM1/4 (D-5, sc-365677, IB 1/1000) antibodies were purchased from Santa Cruz Biotechnology. Alexa Fluor-conjugated anti-TOMM20 mouse monoclonal antibody (ab309166, IF 1/250), Alexa Fluor-conjugated donkey polyclonal anti-goat IgG (ab150130, IF 1/20000), anti-mouse IgG (ab150105, IF 1/20000), and anti-rabbit IgG (ab150073, IF 1/20000) secondary antibodies were purchased from abcam. All antibodies were used at the concentrations recommended by their manufacturers.

Validation

All antibodies used in this study are commercial and validated by the manufacturers. The validation can be found on the manufacturer's websites or in online databases.
anti-EEA1 (C45B10, #3288) antibody, https://www.cellsignal.com/products/primary-antibodies/eea1-c45b10-rabbit-mab/3288
anti-Hexokinase I (C35C4, #2024) antibody, https://www.cellsignal.com/products/primary-antibodies/hexokinase-i-c35c4-rabbit-mab/2024
anti-GAPDH (D16H11, #5174) antibody, https://www.cellsignal.com/products/primary-antibodies/gapdh-d16h11-xp-rabbit-mab/5174
anti-PKM 1/2 (C103A3, #3190) antibody, https://www.cellsignal.com/products/primary-antibodies/pkm1-2-c103a3-rabbit-mab/3190
anti-PKM2 (D78A4, #4053) antibody, https://www.cellsignal.com/products/primary-antibodies/pkm2-d78a4-xp-rabbit-mab/4053
anti-pY849 PDGFRa/pY857PDGFRb (C43E9, #3170) antibody, https://www.cellsignal.com/products/primary-antibodies/phospho-pdgf-receptor-a-tyr849-pdgf-receptor-b-tyr857-c43e9-rabbit-mab/3170
anti-AKT (C67E7, #4691) antibody, https://www.cellsignal.com/products/primary-antibodies/akt-pan-c67e7-rabbit-mab/4691
anti-pT308 AKT (D25E6, #13038) antibody, https://www.cellsignal.com/products/primary-antibodies/phospho-akt-thr308-d25e6-xp-rabbit-mab/13038
anti-ERK1/2 (137F5, #4695) antibody, https://www.cellsignal.com/products/primary-antibodies/p44-42-mapk-erk1-2-137f5-rabbit-mab/4695
anti-phospho ERK1/2 (D13.14.4E, #4370) antibody, https://www.cellsignal.com/products/primary-antibodies/phospho-p44-42-mapk-erk1-2-thr202-tyr204-d13-14-4e-xp-rabbit-mab/4370
anti-GLUT1 (ab115730) antibody, https://www.abcam.com/en-mc/products/primary-antibodies/glucose-transporter-glut1-antibody-epr3915-ab115730#
anti-Hexokinase II (ab209847) antibody, https://www.abcam.com/en-hk/products/primary-antibodies/hexokinase-ii-antibody-epr20839-ab209847
anti-PFKL (ab181064) antibody, https://www.abcam.com/en-hk/products/primary-antibodies/pfkm-pfkl-antibody-epr11904-ab181064
anti-PGK1 (ab199438) antibody, https://www.abcam.com/en-ge/products/primary-antibodies/pgk1-antibody-epr19057-ab199438
anti-ENO1/2/3 (ab189891) antibody, https://www.abcam.com/en-ge/products/primary-antibodies/eno1-eno2-eno3-antibody-epr18407-ab189891#
anti-PDGFRalpha antibodies (AF1062) antibody, https://www.rndsystems.com/products/mouse-pdgf-ralpha-antibody_af1062
anti-pS226 GLUT1 antibody (ABN991) antibody, https://www.emdmillipore.com/US/en/product/Anti-phospho-GLUT-1-Antibody-Ser226,MM_NF-ABN991?bd=1
anti-phosphotyrosine monoclonal antibody cocktail 4G10 Platinum (05-1050) antibody, https://www.emdmillipore.com/US/en/product/4G10-Platinum-Anti-Phosphotyrosine-Antibody-mouse-monoclonal-cocktail-IgG2b,MM_NF-05-1050?bd=1
anti-ALDOA antibody cocktail (711764) antibody, https://www.thermofisher.com/antibody/product/Aldolase-A-Antibody-Recombinant-Polyclonal/711764
polyclonal anti-PDGFRbeta (sc-1627) antibody, https://www.citeab.com/antibodies/821961-sc-1627-pdgfr-antibody-m-20
anti-EEA1 (sc-6414) antibody, https://www.citeab.com/antibodies/790654-sc-6414-eea1-antibody-c-15
anti-clathrin heavy chain (sc-6579) antibody, https://www.citeab.com/antibodies/812414-sc-6579-clathrin-hc-antibody-c-20
anti-dynamin II (sc-6400) antibody, https://www.citeab.com/antibodies/786684-sc-6400-dynamin-ii-antibody-c-18
anti-PDGFRbeta (sc-432) antibody, https://www.citeab.com/antibodies/821960-sc-432-pdgfr-antibody-958

anti-SHP2 (B-1, sc-7384) antibody, https://www.scbt.com/p/sh-ptp2-antibody-b-1
anti-TPI1 (H-11, sc-166785) antibody, https://www.scbt.com/p/tim-antibody-h-11
anti-GPI (H-10, sc-365066) antibody,https://www.scbt.com/p/gpi-antibody-h-10
anti-PGAM1/4 (D-5, sc-365677) antibody, https://www.scbt.com/p/pgam1-4-antibody-d-5
Alexa Fluor-conjugated anti-TOMM20 mouse monoclonal antibody (ab309166) antibody, https://www.abcam.com/en-hk/products/primary-antibodies/alexa-fluor-647-tomm20-antibody-epr15581-39-mouse-igg1-ab309166
Alexa Fluor-conjugated donkey polyclonal anti-goat IgG (ab150130) antibody, https://www.abcam.com/en-am/products/secondary-antibodies/donkey-goat-igg-h-l-alexa-fluor-555-ab150130
Alexa Fluor-conjugated donkey anti-mouse IgG (ab150105) antibody,https://www.abcam.com/en-al/products/secondary-antibodies/donkey-mouse-igg-h-l-alexa-fluor-488-ab150105
Alexa Fluor-conjugated donkeyanti-rabbit IgG (ab150073) antibody,https://www.abcam.co.jp/products/secondary-antibodies/donkey-rabbit-igg-hl-alexa-fluor-488-ab150073.html

Additionally, following antibodies were validated in this study or in a previous publication for immunoblotting and/or immunofluorescence;
anti-Hexokinase I antibody (C35C4, #2024) , validated using siRNAs in Extended Data Fig. 2
anti-GAPDH antibody (D16H11, #5174), validated using an siRNA in Extended Data Fig. 2
anti-PKM 1/2 antibody (C103A3, #3190), validated using siRNAs in Extended Data Fig. 2
anti-GLUT1 antibody (ab115730), validated using siRNAs in Extended Data Fig. 2
anti-Hexokinase II antibody (ab209847), validated using siRNAs in Extended Data Fig. 2
anti-PGK1 antibody (ab199438), validated using siRNAs in Extended Data Fig. 2
anti-ENO1/2/3 antibody (ab189891), validated using siRNAs in Extended Data Fig. 2
anti-ALDOA antibody cocktail (711764), validated using siRNAs in Extended Data Fig. 2
anti-dynamin II antibody (sc-6400), validated using conditional KO cells in Fig. 3e
anti-SHP2 antibody (B-1, sc-7384), validated using conditional KO cells in Tsutsumi et al, Nat. Commun. 8, 466, 2017
anti-TPI1 antibody (H-11, sc-166785), validated using siRNAs in Extended Data Fig. 2
anti-GPI antibody (H-10, sc-365066), validated using siRNAs in Extended Data Fig. 2
anti-PGAM1/4 antibody (D-5, sc-365677) , validated using siRNAs in Extended Data Fig. 2
anti-PDGFRalpha antibodies (AF1062), validated using siRNAs in Extended Data Fig. 5
anti-PDGFRbeta antibodies (sc-1627), validated using siRNAs in Extended Data Fig. 5

Eukaryotic cell lines

Policy information about cell lines and Sex and Gender in Research

Cell line source(s)	Swiss 3T3 (JCRB9019), JCRB Cell Bank (National Institutes of Biomedical Innovation, Health and Nutrition, Japan)<br>Dynamin TKO MEFs, Professor DeCamilli (Yale University)<br>HeLa cells, Sakai Lab stock
Authentication	Swiss 3T3 and Dynamin TKO MEFs were not authenticated after receipt.<br>HeLa cells were not authenticated.
Mycoplasma contamination	All cells were confirmed as mycoplasma-negative by the PCR method.
Commonly misidentified lines<br>(See ICLAC register)	No commonly misidentified cell lines were used in the study

Plants

Seed stocks	*Report on the source of all seed stocks or other plant material used. If applicable, state the seed stock centre and catalogue number. If plant specimens were collected from the field, describe the collection location, date and sampling procedures.*
Novel plant genotypes	*Describe the methods by which all novel plant genotypes were produced. This includes those generated by transgenic approaches, gene editing, chemical/radiation-based mutagenesis and hybridization. For transgenic lines, describe the transformation method, the number of independent lines analyzed and the generation upon which experiments were performed. For gene-edited lines, describe the editor used, the endogenous sequence targeted for editing, the targeting guide RNA sequence (if applicable) and how the editor was applied.*
Authentication	*Describe any authentication procedures for each seed stock used or novel genotype generated. Describe any experiments used to assess the effect of a mutation and, where applicable, how potential secondary effects (e.g. second site T-DNA insertions, mosiacism, off-target gene editing) were examined.*
